# Hyperlipidemia Increases Nalbuphine Brain Accumulation with Multiple Dosing without Affecting Its Analgesic Response—Its Respiratory Depression Potential Should Be Investigated in Future Studies

**DOI:** 10.3390/ph17030282

**Published:** 2024-02-22

**Authors:** Marwa E. Elsherbiny, May Almukainzi, Eman Amer, Marwan Emara

**Affiliations:** 1 Department of Pharmacology and Toxicology, Faculty of Pharmacy, Ahram Canadian University, 6th of October City, Giza 12566, Egypt; marwae@ualberta.ca; 2 Department of Pharmaceutical Sciences, College of Pharmacy, Princess Nourah bint Abdulrahman University, P.O. Box 84428, Riyadh 11671, Saudi Arabia; 3 Department of Biochemistry, Faculty of Pharmacy, Ahram Canadian University, 6th of October City, Giza 12566, Egypt; dr_eman_amer@yahoo.com; 4 Center for Aging and Associated Diseases, Zewail City of Science, Technology, and Innovation, Giza 12578, Egypt; memara@zewailcity.edu.eg

**Keywords:** nalbuphine, hyperlipidemia, pharmacokinetics, pharmacodynamics, drug accumulation, HDL

## Abstract

Nalbuphine is associated with a significant risk of respiratory depression. Its central nervous system entry is hindered by P-glycoproteins, and lower P-glycoprotein activity is a risk factor for respiratory depression. We assessed the effect of hyperlipidemia on nalbuphine pharmacokinetics, brain and liver uptake, and analgesic response following single (2.5 mg/kg) and multiple (2.5 mg/kg/day for three days) doses in normolipidemic and hyperlipidemic rats. Trends of reduction and increase in nalbuphine C_max_ and Vd_ss_/F were observed, respectively, in hyperlipidemic rats. Negative correlations were observed between C_max_ and serum lipoproteins. Serum-normalized brain and liver levels at 1 h post-dose were lower in hyperlipidemic rats, with brain and liver levels being negatively and positively correlated with TG and HDL, respectively. At steady state, marked nalbuphine accumulation was observed in hyperlipidemic rat brains (R = 1.6) compared with normolipidemic rats (R = 1.1). Nalbuphine analgesic response was not altered by hyperlipidemia at steady state. Caution should be exercised since greater brain accumulation in hyperlipidemic patients treated with nalbuphine could increase their risk of respiratory depression. Our study highlights an unexpected role of lipoproteins in drug absorption and tissue uptake. We also propose a model for reduced nalbuphine absorption based on interaction with intestinal HDL-3.

## 1. Introduction

**Figure 1:**
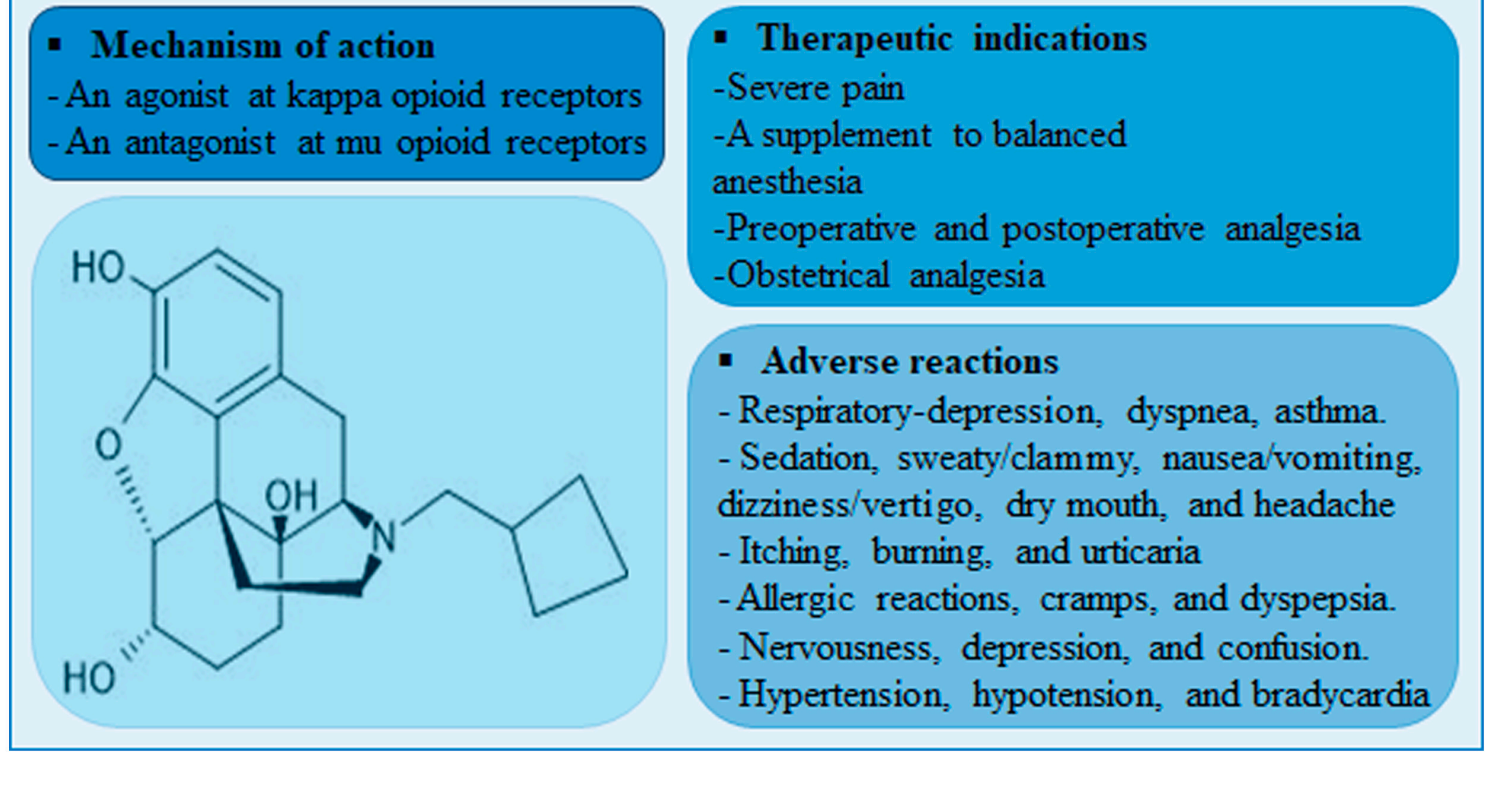
A diagram showing nalbuphine chemical structure, therapeutic indications, mechanisms of action, and adverse reactions.

Nalbuphine is a mixed agonist-antagonist (at κ-receptor and μ-receptor, respectively) opioid analgesic that is indicated for severe pain control. Unlike μ-receptor opioid agonists, nalbuphine is associated with lower incidence of μ-receptor-mediated adverse effects such as pruritis, urinary retention, tolerance, and dependence. Although nalbuphine possesses a ceiling effect at doses > 30 mg, the risk of respiratory depression remains significant [1,2]. A meta-analysis of fifteen randomized clinical trials comparing the analgesic effect and safety profile of nalbuphine and morphine showed that nalbuphine provides analogous analgesia with a lower relative risk of adverse effects including respiratory depression [3]. A summary of nalbuphine’s therapeutic indications, molecular structure, mechanism of action and potential side-effects are presented in Figure 1.

Onset of nalbuphine analgesia is quick ~3 min after intravenous administration and ~15 min after subcutaneous administration [4]. Nalbuphine is a flow-limited drug with an extraction ratio of 0.5–0.7 and poor oral bioavailability (F = 12%) due to extensive first pass metabolism [5,6,7]. Its plasma half-life is approximately 3–6 h in adults with normal hepatic and renal function [2,4,6]. After bolus intravenous administration, plasma concentrations of nalbuphine decline in a biexponential fashion [5]. Nalbuphine is cleared mainly by metabolism in the liver, with ~75% being metabolized by UDP-glucuronosyltransferases and ~25% being metabolized by cytochrome P450 enzymes [8]. Thereafter, nalbuphine and its conjugate metabolites are excreted in feces via bile [9].

Reports suggest that nalbuphine is a P-glycoprotein substrate [10,11,12]. It has a partial degree of penetrance through the blood brain barrier due to P-glycoprotein-mediated efflux [13]. In fact, a change in P-glycoprotein activity is considered a risk factor for opioid-induced respiratory depression in pediatrics [14]. One of the factors that could alter P-glycoprotein activity is hyperlipidemia [15,16]. Increased lipoprotein levels were shown to decrease P-glycoprotein expression/activity, increase tissue uptake, and decrease clearance of P-glycoprotein substrates such as cyclosporin A [15,16,17]. Based on the facts that nalbuphine is a P-glycoprotein substrate and that hyperlipidemia was shown to reduce P-glycoprotein activity, it is plausible that nalbuphine brain entry, and consequently its pharmacological effects, could be enhanced in hyperlipidemic patients.

According to the World Health Organization, the global prevalence of elevated total cholesterol was ~39% among adults in 2008 [18,19]. Based on the high prevalence of increased cholesterol levels and the greater risk of opioid-induced respiratory depression with reduced P-glycoprotein activity, we hypothesize that hyperlipidemia boosts brain nalbuphine uptake and therefore could enhance its pharmacological effects in this patient population. Herein we assess the effect of high lipoprotein levels on nalbuphine pharmacokinetics, brain and liver uptake, and analgesic response following single and multiple doses. We used the single-dose poloxamer 407 (P407) rat model of hyperlipidemia [20]. This model has been extensively used in hyperlipidemia research mainly because it produces marked hyperlipidemia (within 24 h) via inhibition of lipoprotein lipase and indirect stimulation of 3-hydroxy-3-methylglutaryl coenzyme A [15,17,20]. It should be noted that several reports have shown an absence of poloxamer-induced hepatotoxicity when poloxamer is administered in a single-dose regimen in contrast to the chronic poloxamer 407 model of hyperlipidemia [21,22,23,24,25,26,27]. After 1 mg/kg i.p. injection of poloxamer 407, the C_max_ of triglycerides (TG) and total cholesterol occurs at 36 h post-dose [20]. However, it is recommended that studies be initiated at time points later than 36 h if similar elevations in lipoprotein levels to those observed in humans are of interest [20]. Our results show that increased lipoproteins reduce nalbuphine absorption rate, but not extent, and alter its tissue uptake following a single-dose regimen. We provide associative evidence of involvement of lipoproteins in nalbuphine transport. Following multiple nalbuphine doses, increased lipoproteins are associated with greater serum-normalized brain and liver concentrations at 1-h post-nalbuphine dose, greater nalbuphine accumulation and delayed serum brain tissue equilibration, however, these changes do not seem to alter its analgesic response. 

## 2. Results

### 2.1. Single-Dose Pharmacokinetic Study

**Figure 2:**
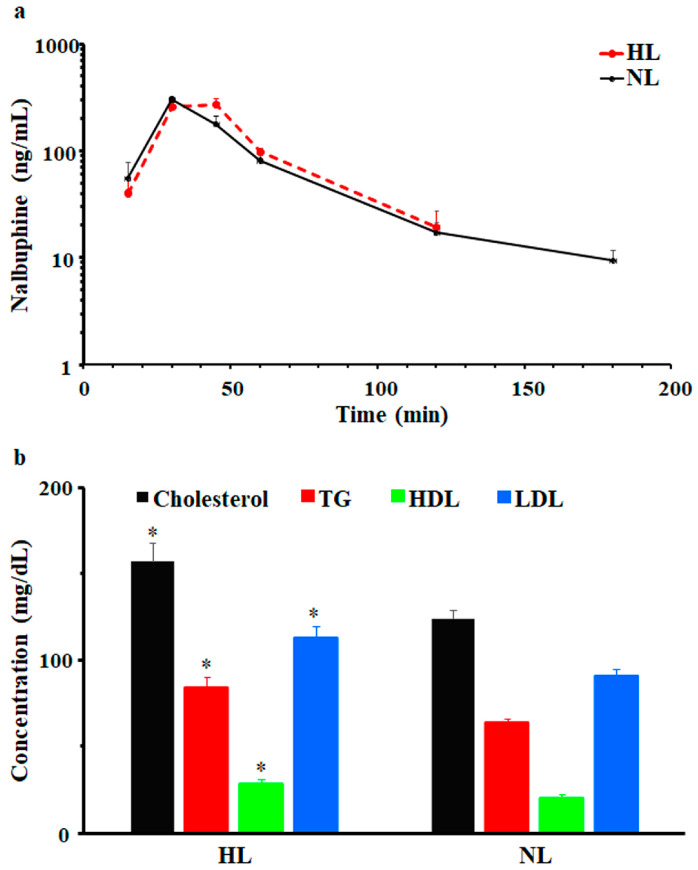
(**a**) Mean ± SE nalbuphine serum concentration–time profiles in rats after a single intraperitoneal dose of 2.5 mg/kg nalbuphine given to normolipidemic (NL) or hyperlipidemic (HL) rats, (**b**) Poloxamer 407 induced significant increases in total cholesterol, triglycerides (TG), high-density lipoprotein (HDL), and low-density lipoprotein (LDL) in HL rats compared to NL rats. * indicates significant difference from NL rats (*p* < 0.05).

After a single dose of 2.5 mg/kg i.p., nalbuphine was first detected at 15 min and C_max_ was observed at 30 min in NL and HL rats; serum concentrations vs. time profiles are shown in Figure 2a. Beyond 180- and 120-min post-dose, nalbuphine serum levels were undetectable in NL and HL sera, respectively. Generally, serum nalbuphine concentrations and pharmacokinetic parameters were not significantly different between HL and NL rats; however, as shown in Figure 2a and Table 1, serum nalbuphine concentrations vs. time curve seemed to be shifted slightly to the right in HL rats, indicating slower absorption with a bit lower C_max_ (15.5%) and higher Vd_ss_/F (47.5%). Serum lipid analysis confirmed the validity of the model, since significant elevations in total cholesterol (27%), TG (32%), LDL (24%), and HDL (40%) levels were observed, as shown in Figure 2b.

**Figure 3:**
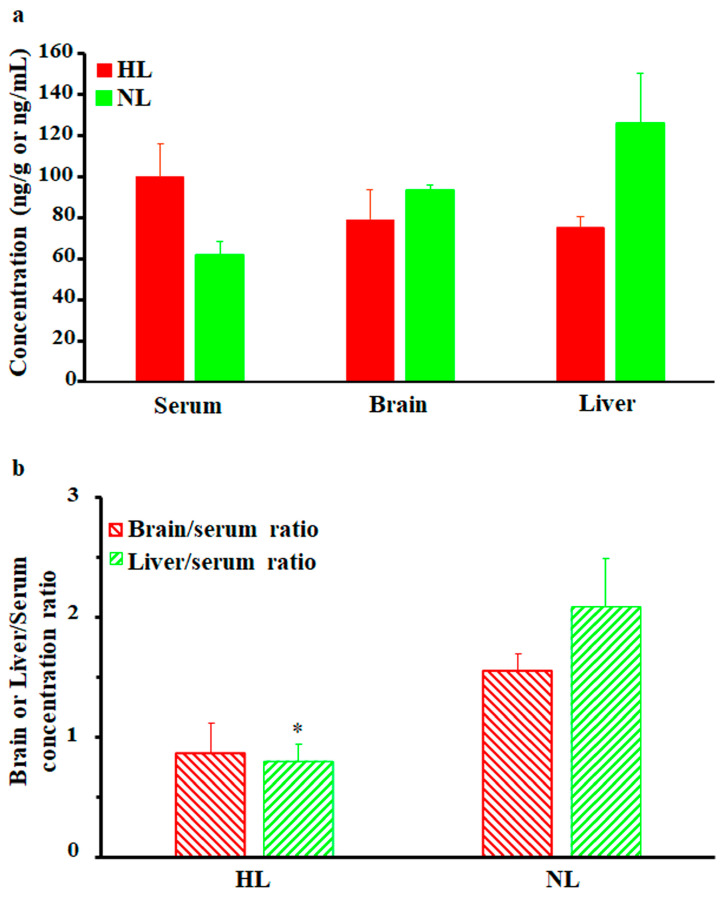
(**a**) Mean ± SE nalbuphine brain and liver concentrations in rats 1 h after a single intraperitoneal dose of 2.5 mg/kg nalbuphine given to normolipidemic (NL) or hyperlipidemic (HL) rats (*n* = 3/group). (**b**) Brain/serum and liver/serum concentration ratios in HL and NL rats 1 h after a single intraperitoneal dose of 2.5 mg/kg nalbuphine. * indicates significant difference from NL rats (*p* < 0.05).

As shown in Figure 3a, brain and liver nalbuphine concentrations at 1 h post-dose seemed lower in HL rats than NL rats by 15% and 40%, respectively, but statistical significance was not achieved. Serum-normalized levels, shown in Figure 3b, reproduced this lower nalbuphine trend in HL brain (by 44%, *p* = 0.079) and liver (by 62%, *p* = 0.039) compared with NL tissues. Because of the marked variability associated with nalbuphine levels, we performed Pearson correlation analysis between nalbuphine serum C_max_/brain/liver concentrations and serum lipid concentrations. In line with our earlier results, significant negative correlations were observed between serum nalbuphine C_max_ and serum total cholesterol, HDL, and LDL levels, with a similar trend being observed with TG; the Pearson correlation coefficients are shown in Table 2. These results confirm the lowering effect of serum lipids on nalbuphine absorption rate, especially showing that when Pearson correlation analysis was run between nalbuphine serum levels at 1 h post-dose and serum lipids, no correlation was observed. Furthermore, a strong negative correlation between brain nalbuphine levels at 1 h and serum TG levels was observed with the absence of any correlation with other serum lipids, as shown in Table 2. By contrast, a strong positive correlation trend (*p* = 0.0767) was observed between the 1 h liver nalbuphine levels and serum HDL concentrations. These findings indicate that serum lipids modify nalbuphine tissue uptake with TG-lowering and HDL-increasing brain and liver tissue uptake, respectively.

### 2.2. Multiple-Dose Tissue Distribution Study

**Figure 4:**
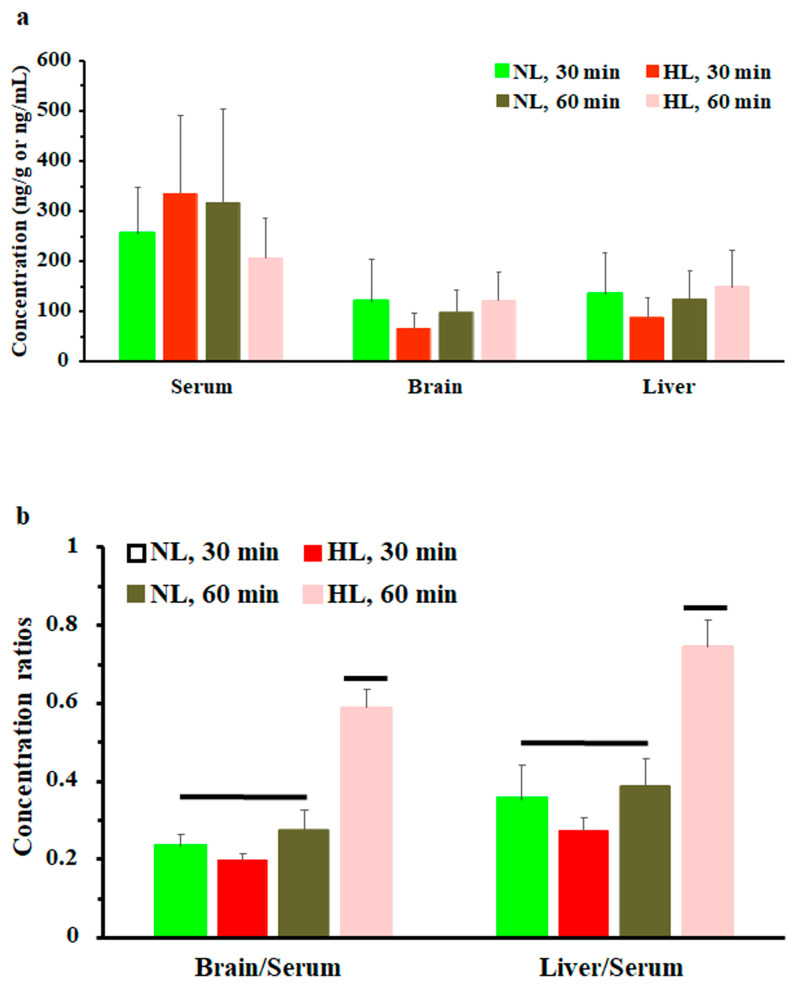
(**a**) Nalbuphine serum, brain, and liver concentrations in rats 30 and 60 min after the third intraperitoneal 2.5 mg/kg daily dose of nalbuphine given to normolipidemic (NL) or hyperlipidemic (HL) rats (*n* = 4/group). (**b**) Brain/serum and liver/serum concentration ratios in HL and NL rats 30 and 60 min after the third intraperitoneal dose of 2.5 mg/kg/day nalbuphine. Data presented as mean ± SE, and groups under separate lines are significantly different, *p* < 0.05.

**Figure 5:**
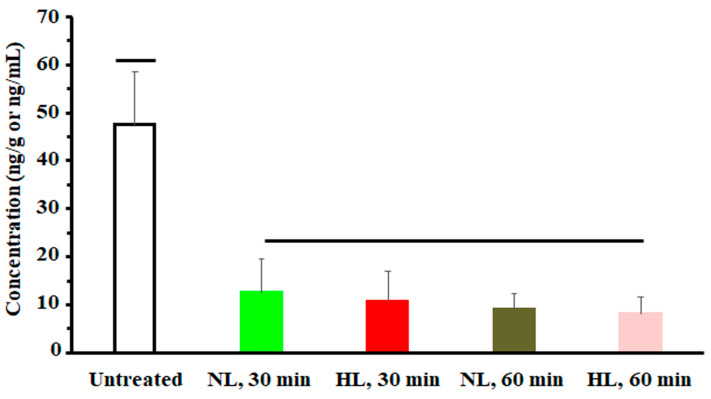
A plot of nalbuphine analgesic response (indicated by number of writhes) in NL and HL rats 30 and 60 min after the third intraperitoneal dose of 2.5 mg/kg nalbuphine. One-way analysis of variance was used to assess the significance of difference. Groups under separate lines are significantly different, *p* < 0.05.

To assess the effect of hyperlipidemia on nalbuphine steady state levels, nalbuphine was administered daily for three days. Figure 4a shows that nalbuphine serum, brain, and liver concentrations were not significantly different between HL and NL rats at any of the tested time points. However, serum-normalized brain and liver concentration ratios at 60 min, shown in Figure 4b, were significantly higher in HL rats than in the rest of the groups (*p* = 0.0001 and 0.0028, respectively). Notably, serum-normalized brain and liver nalbuphine levels equilibrated quickly in NL rats, since these ratios were similar at 30- and 60-min post-dose (0.24 and 0.27 for brain and 0.36 and 0.39 for liver, respectively), as shown in Figure 4b. However, brain/serum and liver/serum concentration ratios increased from 0.20 and 0.27 at 30 min to 0.59 and 0.75 at 60 min, respectively, in HL rats, indicating longer equilibration time and ~2-fold greater serum-normalized brain and liver levels in HL rats with repeated nalbuphine administration, as shown in Figure 4b. Serum nalbuphine levels did not show any accumulation in NL rats with repeated doses (R = 0.85) but a relatively marked accumulation was seen in HL rats (R = 1.32). When these ratios were calculated using nalbuphine 1-h levels in the brain and liver, similar findings were observed. Brain and liver nalbuphine levels did not show any accumulation in NL rats with repeated doses (R = 1.1 and 1.0, respectively), but a relatively marked accumulation was seen in HL rats (R = 1.6 and 1.63, respectively). Nalbuphine analgesic response was not altered in HL rats compared with NL rats at 30- and 60-min post-dose, Figure 5, indicating that at this dose level hyperlipidemia-induced changes in nalbuphine absorption rate, serum-normalized brain tissue levels and delayed equilibration, and accumulation do not alter nalbuphine’s therapeutic response.

## 3. Discussion

Nalbuphine is the only mixed agonist opioid analgesic that is not controlled under the Controlled Substances Act and is primarily used for the management of moderate to severe pain in hospitals [28]. Although its annual prescription has declined from 14,803 prescriptions in 2016 to 951 prescriptions in 2020 in the United States, it increased again in 2021 with a total prescription count of 4389 [28]. Nalbuphine was approved for marketing in the United States in 1979; however, little is known about factors affecting its pharmacokinetics and pharmacodynamics. In this article we show, based on associative evidence and reduced serum-normalized tissue concentrations, that hyperlipidemia slightly decreases nalbuphine absorption rate and increases its distribution volume, with evidence of the contribution of lipoproteins to nalbuphine tissue uptake. At steady state, hyperlipidemia increased serum-normalized brain and liver concentrations at 1 h post-nalbuphine dose, delayed brain and liver tissue equilibration, and was associated with greater accumulation in serum and in both tested tissues; however, these changes had no effect on nalbuphine analgesic response. 

Nalbuphine pharmacokinetics have been previously reported in rats and mice [8,29]. In rats, T_max_ ranges from 15–30 min after oral administration, which agrees with our observation after intraperitoneal dosing [8,30]. Nalbuphine T_1/2_ is shorter after intravenous than oral dosing, with reported average values of 56 min and 120 min, respectively, which is potentially caused by the enterohepatic recycling of the drug [8,31,32]. We observed T_1/2_ values that ranged from 18–42 min after intraperitoneal dosing, which are closer to those reported after intravenous administration in rats and those reported after intraperitoneal administration in mice (56.4 min) [29]. Blood CL and Vdss of 38.7 mL/min and 1917 mL, respectively, were reported after intravenous administration in rats, and plasma CL/F and Vdz/F of 27,901 mL/h/kg and 37,930 mL/kg, respectively, were reported after intraperitoneal administration in mice [29,31]. This blood clearance value of nalbuphine in rats is slightly greater than the total average hepatic blood flow in rats (35.4 mL/min) [33], indicating contribution of other organs to the clearance of nalbuphine. In line with this presumption, we observed that the previously reported apparent nalbuphine plasma clearance of 27,901 mL/h/kg after intraperitoneal administration in mice far exceeds the normal hepatic blood flow of mice (~2 mL/min for an average 20 g mouse) even after correction for plasma hepatic flow [HBF = HPF/(1 − hematocrit)] and performance of the per kg calculation (56.5 mL/min/kg) [34]. Indeed, apparent nalbuphine plasma clearance values following intraperitoneal doses are still compounded by the factor of bioavailability. Nalbuphine bioavailability ranges from <1% to 2.7% following oral administration in rats [30,32]. Based on our results, it seems that nalbuphine bioavailability is markedly increased after intraperitoneal dosing compared with oral dosing considering the previously reported values of rat blood clearance and bioavailability [30,31,32].

Although drug lipoprotein interactions were characterized a long time ago, only relatively recent reports acknowledge the modulation of drug pharmacokinetics by lipoproteins [35,36]. For example, marked increases in plasma levels of amiodarone and cyclosporine A along with significant reductions in their Vd and plasma unbound fraction were previously noted in hyperlipidemic rats [17,37]. By contrast, evidence of LDL-mediated hepatic uptake of other drugs does exist, such as clopidogrel (from in vitro and in vivo experiments) and ticlopidine (from in vitro experiments) [36]. Herein, nalbuphine pharmacokinetics were not altered by hyperlipidemia except for a minor reduction in absorption rate, indicated by reduced average C_max_ (by 15.5%), and increased apparent steady state volume of distribution, indicated by a 47.5% increase in the average Vd_ss_/F value. 

**Figure 6:**
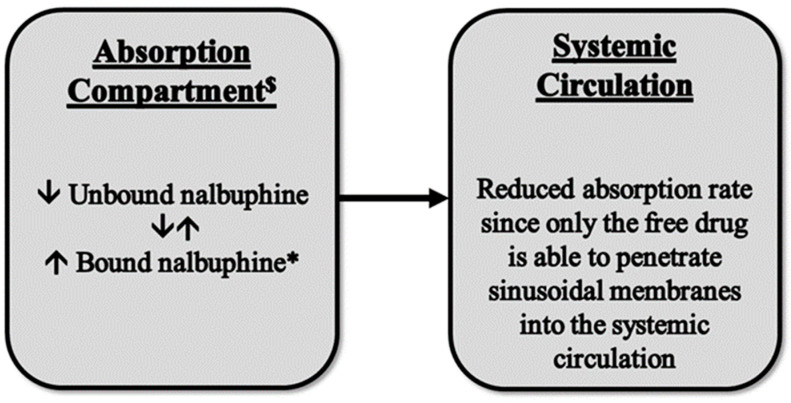
A proposed model for the observed reduction in nalbuphine absorption rate in HL rats. $ Absorption compartment: mesenteric veins, hepatic portal vein, and hepatic tissue. * Nalbuphine HDL3 bound form.

Although changes in nalbuphine C_max_ were not statistically significant due to the large variability, the reduced nalbuphine absorption rate was confirmed by significant negative correlations between its C_max_ and serum lipoproteins, with a strong negative correlation being observed between C_max_ and HDL levels (r = −0.712). We have not found a similar observation reported for any drug thus far and, therefore, the available literature does not provide an explanation for this finding. However, it has been reported that plasma protein binding of nalbuphine is ~50% [38]. Thus, the association of nalbuphine with lipoprotein particles and reduced free drugs in the portal circulation might have hindered drug entry into the systemic circulation; a proposed model is shown in Figure 6. In support of this assumption, it has been shown that the small intestine can make HDL-3, a subspecies of HDL, in addition to chylomicrons. However, unlike chylomicrons, HDL-3 travels through the portal vein instead of the lymphatics [39]; therefore, increased nalbuphine-HDL3 association and decreased free nalbuphine could explain the reduced absorption rate, especially because the correlation between nalbuphine C_max_ and HDL was the strongest among all the other lipoproteins. It is worth noting that based on our observation of a strong positive correlation between liver nalbuphine levels and HDL, shown in Table 2, it is likely that greater hepatic nalbuphine extraction occurs in HL rats during first pass, especially considering that SR-B1 is heavily expressed in liver sinusoidal endothelial cells [40]. This assumption agrees with both observations of strong negative and strong positive correlations between nalbuphine serum C_max_ and HDL, and nalbuphine liver concentration and HDL, respectively.

The higher average value of Vd_ss_/F and the lower serum-normalized brain and liver concentrations indicate a greater nalbuphine distribution volume in HL rats compared with NL controls and suggest potential involvement of lipoproteins in nalbuphine transport and tissue uptake. In support of this assumption, we observed a strong negative correlation between brain nalbuphine concentrations at 1 h and serum TG levels. TG-rich lipoproteins (chylomicrons and VLDL) transport TG to muscle and adipose tissue cells for fatty acid release and subsequent use in energy production or fat storage, respectively [41]. Thus, it is possible that nalbuphine is transported along in these lipoproteins to these tissues in HL rats. Similarly, in support of lipoproteins’ modulation of nalbuphine tissue uptake, we observed a strong positive correlation (*p* = 0.0767) between the 1-h liver nalbuphine concentrations and HDL levels. HDL is indispensable in reverse cholesterol transport from peripheral tissues to the liver and it also interacts with the TG-rich lipoprotein, VLDL, to facilitate its conversion to LDL [42]. The positive correlation between HDL levels and nalbuphine hepatic levels indicates that HDL plays an important role in nalbuphine liver uptake, either directly through reverse cholesterol transfer or indirectly through LDL receptor-mediated endocytosis of LDL particles.

Our findings on the lipoprotein modulation of drug transport and tissue uptake agree with the findings reported by Hideaki Yamamoto et al. 2017 [36]. The authors showed that drugs such as amiodarone and ticlopidine display increased association with VLDL in Triton WR-1339-induced hyperlipidemic rats. In this study, LDL receptor overexpression in mouse liver hepatoma cells resulted in increased uptake of lipoproteins associated drugs such as ticlopidine and ticagrelor. The contribution of the LDL receptor to the uptake and transport of these drugs was further confirmed in an LDL receptor knock-down human-derived hepatic cell line and in familial hypercholesterolemic patients undergoing lipoprotein apheresis treatment [36].

Since cases of repeated nalbuphine administration are likely, we decided to assess nalbuphine tissue uptake, specifically into the brain and liver, and accumulation at steady state in HL rats. Upon repeated exposure, we did not observe any trend of reduced absorption at the observed T_max_ (30 min), which was likely caused by the greater accumulation seen in HL rats compared with NL controls. In healthy humans, repeated oral administration of nalbuphine has been associated with modest accumulation (R = 1.6) which, considering our findings, might be enhanced in hyperlipidemic patients [38]. Therefore, caution might be essential when repeated nalbuphine dosing is needed in patients with elevated lipoprotein levels, as the outcomes of an even greater extent of accumulation in these patients are unclear. Equilibration between serum and both of the tested tissues (brain and liver) was delayed in HL rats in the multiple dose study. This finding further supports our initial observation of increased nalbuphine distribution volume in HL rats. To test whether this delay in brain tissue equilibration would delay nalbuphine analgesic response, we performed the writhing test at two time points (30 and 60 min). Nalbuphine was equally effective at both time points and its analgesic effect in HL rats matched that observed in NL controls. 

Our findings suggest the following: (1) potentially reduced analgesic response because of the reduced absorption rate when nalbuphine is administered acutely as a single dose via non intravenous routes and (2) greater serum-normalized brain levels and increased nalbuphine accumulation, not only in the circulation but also in the brain, the site where the potentially fatal respiratory depression adverse effect might ensue. Indeed, our study has several limitations, which include the use of the rat as a model, which may not accurately represent humans, especially considering that the single-dose poloxamer model produces quick onset hyperlipidemia, which does not mimic the slow chronic process in humans [20]. Another limitation is the marked increases (for up to 36 h post-poloxamer injection) in different lipoprotein levels yielded by the model used in our study, which do not represent elevations seen in hyperlipidemic patients. However, it is recommended that the model be used after 36 h post-poloxamer injection if clinically relevant elevations in lipoprotein levels are of interest [20]. Accordingly, to overcome this limitation, we started nalbuphine injection at 65 h post-poloxamer injection.

## 4. Materials and Methods

### 4.1. Animals

Inhouse-bred adult male Sprague Dawley rats from Ahram Canadian University animal house with average weights of 176 ± 21.8 g were used in all studies. The animals were kept in cages in the animal facility at the university, under constant temperature (~23 °C) and humidity (~50%). They were permitted access to a standard rodent diet and water ad libitum. The study was approved by the Faculty of Pharmacy-Ahram Canadian University research ethics committee (Protocol number: REC 0423) and all experimental work was conducted at the Ahram Canadian University.

### 4.2. Single-Dose Pharmacokinetic Study

The rats were allocated into two groups (*n* = 16, 8 rats/group), normolipidemic (NL) and hyperlipidemic (HL) groups. Approximately 65 h before the pharmacokinetics study, HL rats were rendered hyperlipidemic by intraperitoneal administration of 1 g/kg poloxamer 407 (0.13 g/mL solution in normal saline). At this time, NL rats received 1 mL/kg saline via the same route. Nalbuphine HCl (20 mg/mL vial, SPIMACO MISR for Pharmaceutical Industries) was diluted 1:10 in sterile saline and was administered at 2.5 mg/kg intraperitoneally to all rats. Thereafter, ~300 μL blood was collected by retroorbital bleeding at 5, 10, 15, 30, 45, 60, 120, 180, and 220 min for nalbuphine serum concentration analysis. Blood withdrawal was followed by intraperitoneal injection of an equivalent volume of saline. Three rats from each group were sacrificed at 1 h and serum, brain, and liver tissues were collected to assess tissue distribution after the single nalbuphine dose. Serum samples were also used for analysis of lipoprotein content. Serum and tissue samples were kept at −20 °C until analysis.

### 4.3. Multiple-Dose Tissue Distribution Study

To assess the effect of hyperlipidemia on nalbuphine serum levels and tissue distribution, especially in the brain and liver at steady state, we conducted a multiple-dose study. Rats were allocated into four groups (*n* = 16, 4 rats/group): two groups were normolipidemic (NL) and the other two groups were hyperlipidemic (HL). These groups were labeled as follows: NL (30 min), NL (60 min), HL (30 min), and HL (60 min), with time of sacrifice indicated between parentheses. In this study, hyperlipidemia was induced as described under the single-dose pharmacokinetic study but nalbuphine was given at 2.5 mg/kg/day intraperitoneally for three consecutive days. After the third dose, NL and HL rats were sacrificed at 30 min and 60 min and serum, brain, and liver samples were collected and kept at −20 °C for nalbuphine analysis. Nalbuphine analgesic response was also assessed in these rats before sacrifice at 30 and 60 min after the third nalbuphine dose using the acetic acid-induced writhing test, as previously described [43,44,45]. Each rat was injected with acetic acid solution (0.6% *v*/*v*, 10 mL/kg, i.p.) at 30 and 60 min post-nalbuphine dose. Subsequently, the number of writhes (indicated by abdominal and/or hindlimb stretches) was recorded for each rat over a 30-min observation period.

### 4.4. Serum Lipid Measurement

Serum concentrations of total cholesterol, triglycerides (TG), LDL, and HDL were measured using kits purchased from Egyptian Company for Biotechnology (Spectrum Diagnostics, Cairo, Egypt) according to the manufacturer’s instructions.

### 4.5. Serum and Tissue Sample Preparation 

Brain and liver tissues were excised, and the weight of each tissue was determined. Then the tissues were homogenized (1 g in 5 mL) in acetonitrile: PBS pH 7.4 (70:30) and the homogenate were used for the determination of nalbuphine concentration. The homogenized tissue was then centrifuged at 5000 rpm for 15 min and the supernatant was stored at −80 °C until analysis [46]. Serum samples, 0.2 mL, were diluted with 70% acetonitrile (1:1), centrifuged at 5000 rpm for 15 min, and the supernatant was stored at −80 °C until assayed. At the time of analysis, 0.2 mL of the resulting supernatant was brought up to 1 mL by adding Britton–Robinson buffer solution (pH 6.0).

### 4.6. Nalbuphine Analysis

Nalbuphine concentration was determined using voltametric analysis, employing a previously published method [47]. The analysis was conducted by Dr. Omar Abdel-Hamed Ahmed-Farid, a scientific researcher in the physiology department at the Egyptian National Organization for Drug Control and Research (NODCAR) who was blinded to the experimental design and sample identity. 

Voltametric measurements were obtained using the electrochemical analyzer Computrace system with 797 VA Computrace software (1.0) from Metrohm, Switzerland. A three-electrode cell was employed. A commercial nanogold-based screen-printed carbon electrode (GSPE) with a reference number of DRP-1110 was obtained from DropSens Technology Ltd. (Oviedo, Asturias, Spain). For analytical application, the following parameters were employed: DPV-pulse amplitude 50 mV, pulse width 40 ms, and scan rate 100 mVs^−1^, pulse amplitude 20 mV, potential step 6 mV, and an electrochemical analyzer made the background subtraction automatically. 

In the electrochemical measurements, GSPE was electrochemically pretreated by scanning the potential between 0.02 and 0.30 V with a scan rate of 20 mV s^−1^ for 100 cycles in 0.04 mol L^−1^ Britton–Robinson buffers. Voltametric analyses were performed in 30 µL of Britton–Robinson buffer solution (pH 6.0) containing serum or tissue samples. Calibration curves (from 2.5–300 ng/mL) of nalbuphine using DPV were established by plotting the peak current Iρ (µA) against drug concentration (ng/mL). 

The assay method was validated in blank rat serum spiked with nalbuphine to achieve concentrations of 2.5, 10, 25, 50, 100, 200, and 300 ng/mL in a final volume of 0.2 mL. There were three replicates of each concentration, repeated over three days. These samples were diluted with 70% acetonitrile (1:1) and then centrifuged at 5000 rpm for 15 min. The supernatant, 0.2 mL, was brought up to 1 mL by adding Britton–Robinson buffer solution (pH 6.0) and 30 µL of the final mixture was applied to the GSPE strip and subjected to voltametric analysis as described above. The linearity of the method was confirmed with an R2 of 0.992 ± 0.004 (average ± SD). The mean recovery and both intra- and inter-day coefficients of variation were 98.5% ± 2.5 and <15%, respectively, with LOQ and LOD of 380.8 pg/mL and 126.9 pg/mL, respectively. 

### 4.7. Data Analysis

Data are expressed as mean ± SE, unless otherwise indicated. The noncompartmental approach was used to calculate the pharmacokinetic parameters of apparent clearance (CL/F = Dose/(AUC_0-inf_)), area under serum concentration vs. time curve 0-Tlast (AUC_0-Tlast_), AUC_0-inf_, and apparent volume of distribution at steady state (Vd_ss_/F = ×CL/F) [48]. Nalbuphine serum half-life (t_1/2_) was calculated based on data points (3 points) in the terminal phase with a coefficient of correlation ≥ 0.90. The maximal serum concentration (C_max_) and time to reach C_max_ (T_max_) were read directly from the observed data. The nalbuphine accumulation factor (R) was calculated from the ratio of C_max_ at steady state to C_max_ after the first dose [49].

For statistical comparisons, the F-test was run first to compare sample variances, followed by the appropriate *t*-test for pharmacokinetic parameters, serum concentrations, and serum lipoproteins. One-way analysis of variance (ANOVA) followed by Tukey’s Test for pairwise comparisons were used to assess the significance of differences in nalbuphine levels and analgesic response after multiple doses. F-test and *t*-test were run using Excel Microsoft 365 (Microsoft, Redmond, WA). ANOVA and Pearson correlation analysis between nalbuphine C_max_, brain and liver concentrations, and serum lipid concentrations were performed using SigmaPlot 13 software (Systat Software Inc., Chicago, IL, USA).

## 5. Conclusions

In conclusion, hyperlipidemia reduces the nalbuphine absorption rate, but not its extent, and alters its uptake in the brain and liver after single-dose administration. Following multiple doses, it is associated with increased serum-normalized brain (and liver) levels, a greater extent of nalbuphine accumulation, and delayed serum brain (and liver) tissue equilibration; however, these changes do not alter its analgesic response. Caution should be exercised in dosing nalbuphine in hyperlipidemic patients where its toxicity (especially respiratory depression) might be enhanced by the greater extent of accumulation. Furthermore, it should be noted that some anti-hyperlipidemic drugs possess P-glycoprotein inhibitory effects, besides their lipid-lowering therapeutic effect, such as atorvastatin, lovastatin, and simvastatin [50,51], and potential drug–drug interactions with nalbuphine are indeed possible via multiple mechanisms, which makes the outcome of this interaction hard to predict. Therefore, future studies assessing the outcome of this combination are needed to guide nalbuphine therapy in hyperlipidemic patients.

## Figures and Tables

**Table 1:** Pharmacokinetic parameters (mean ± SE) of nalbuphine in rat serum after intraperitoneal administration of a single 2.5 mg/kg dose in normolipidemic (NL) and hyperlipidemic (HL) rats.

C_max_ (ng/mL)	T_max_ (min)	AUC_0-Tlast_ (ng.min/mL)	AUC_0-inf_ (ng.min/mL)	t_1/2_ (min)	CL/F (mL/min/kg)	Vd_ss_/F (mL/kg)
NL rats						
303 ± 6.7	30	13,307 ± 1850	13,869 ± 1836	26 ± 3.0	195 ± 16.7	2165 ± 315
HL rats						
256 ± 26	30	12,860 ± 1269	13,988 ± 1267	27 ± 3.4	188 ± 15.4	3193 ± 820

The maximal serum concentration (C_max_), time to reach C_max_ (T_max_), area under serum concentration vs. time curve _0-Tlast_ (AUC_0-Tlast_), AUC_0-inf_, serum half-life (t_1/2_), apparent intraperitoneal clearance (CL/F), and apparent intraperitoneal volume of distribution at steady state (Vdss/F). Pharmacokinetic parameters were estimated by noncompartmental analysis; serum half-life (t_1/2_) was calculated based on data points (*n* = 3) in the terminal phase with a coefficient of correlation ≥ 0.90.

**Table 2:** Pearson correlation coefficients between nalbuphine serum/brain/liver concentrations and serum lipid levels.

Correlation of Nalbuphine C_max_ with Different Serum Lipids	
Total cholesterol	r = −0.606→(0.0129) *n* = 16
Triglycerides	r = −0.455→(0.0767) *n* = 16
High-density lipoproteins	r = −0.712→(0.00196) *n* = 16
Low-density lipoproteins	r = −0.568→(0.0217) *n* = 16
Correlation of Brain Nalbuphine Concentration with Different Serum Lipids	
Total cholesterol	r = −0.513→(0.298) *n* = 6
Triglycerides	r = −0.842→(0.0353) *n* = 6
High-density lipoproteins	r = 0.385→(0.451) *n* = 6
Low-density lipoproteins	r = −0.467→(0.350) *n* = 6
Correlation of Liver Nalbuphine Concentration with Different Serum Lipids	
Total cholesterol	r = 0.450→(0.371) *n* = 6
Triglycerides	r = −0.146→(0.782) *n* = 6
High-density lipoproteins	r = 0.764→(0.0767) *n* = 6
Low-density lipoproteins	r = 0.290→(0.578) *n* = 6

r indicates Pearson correlation coefficient, *p*-value is indicated between parentheses, and n is the total number of rats.

## Data Availability

The authors declare that all the data supporting the findings of this study are contained within the paper.

## Abbreviations

C_max_	The maximum serum concentration
T_max_	Time to reach C_max_
AUC_0-Tlast_	Area under serum concentration vs. time curve from 0 to the last time point
AUC_0-inf_	Area under serum concentration vs. time curve from 0 to infinity
t_1/2_	Serum half-life
CL/F	Apparent intraperitoneal clearance
Vd_ss_/F	Apparent intraperitoneal volume of distribution at steady state
NL	Normolipidemic group
HL	Hyperlipidemic group
TG	Triglycerides
HDL	High density lipoprotein
LDL	Low density lipoprotein
VLDL	Very low-density lipoprotein
i.p.	Intraperitoneal injection
r	Pearson correlation coefficient
HBF	Hepatic blood flow
HPF	Hepatic plasms flow
min	Minutes
